# Isotope Ratio Mass Spectrometry (IRMS)-Based Authentication of the Geographic Origins of *Volvariella volvacea* (Bull.) Singer

**DOI:** 10.3390/foods14061074

**Published:** 2025-03-20

**Authors:** Xing Liu, Qinxiong Rao, Qicai Zhang, Hao Geng, Yangyang Lu, Zhu Liu, Shanshan Chen, Peijun Li, Weiguo Song

**Affiliations:** 1 Guangdong Provincial Key Laboratory of Utilization and Conservation of Food and Medicinal Resources in Northern Region, Shaoguan University, Shaoguan 512005, China; liuxinglyg@126.com (X.L.); liuzhu@sgu.edu.cn (Z.L.); 2 Institute for Agro-Food Standards and Testing Technology, Shanghai Academy of Agricultural Sciences, Shanghai 201403, China; qinxiongrao@163.com (Q.R.); qicaizhang@126.com (Q.Z.); genghao0128@163.com (H.G.); yyanglz@163.com (Y.L.); 20151069@saas.sh.cn (S.C.); 3 Shanghai Service Platform of Agro-Products Quality and Safety Evaluation Technology, Shanghai 201403, China; 4 School of Biology & Agriculture, Shaoguan University, Shaoguan 512005, China; 5 School of Food Science & Technology, Shaoguan University, Shaoguan 512005, China

**Keywords:** *Volvariella volvacea*, stable isotope, nutrition component, geographic origin, PLS-DA

## Abstract

The growing consumption of *Volvariella volvacea* has heightened concerns regarding its geographical authenticity. This study analyzed the proteins, 16 common amino acids, and 10 mineral elements (Ca, Cu, Fe, K, Mn, Mg, Na, Se, Sr, Zn) in samples from Fujian, Guangdong, Hubei, Jiangsu, Jiangxi, Shanghai, and Zhejiang, China, along with regional variations in stable isotope ratios. PCA and PLS-DA were applied for origin authentication. The results showed an average protein content of approximately 30 g/100 g (dry basis), with Guangdong samples being the highest. Amino acids exhibited significant regional differences, but the total essential amino acid and total amino acid contents did not. Mineral elements varied significantly by region, except for Cu and K. The Fujian, Hubei, Jiangxi, and Zhejiang samples exhibited significantly higher *δ*^13^C and *δ*^15^N values, while Shanghai samples had significantly higher *δ*^2^H and *δ*^18^O values. These differences enabled PCA to classify the samples into two groups: FHJZ (Fujian, Hubei, Jiangxi, Zhejiang) and GJS (Guangdong, Jiangsu, Shanghai). The PLS-DA model achieved 93.60% accuracy in distinguishing these two groups. Pairwise accuracy within the GJS group exceeded 80%, whereas that within the FHJZ group requires further improvement. These findings support the feasibility of stable isotope analysis for authenticating the geographical origin of *Volvariella volvacea*.

## 1. Introduction

By 2023, the United Nations Food and Agriculture Organization (FAO) reported that around 750 million people worldwide suffered from hunger. Moreover, an estimated 28.9% of the global population (approximately 2.33 billion individuals) experienced varying degrees of food insecurity, ranging from moderate to severe [1]. These alarming figures underscore the growing challenges of food insufficiency and malnutrition, which place significant pressure on traditional agricultural resources and highlight the urgent need for comprehensive solutions to address the underlying causes of global food and nutrition insecurity. In this context, edible mushrooms, as an efficient biological resource, which not only offer significant nutritional value but also effectively utilize agricultural production waste, are being increasingly recognized for their production potential [2]. The FAO reports that global edible mushroom production has exceeded 50 million tons annually [3], further emphasizing the growing importance of edible mushrooms in global food security.

*Volvariella volvacea* (Bull.) Singer, commonly known as the straw mushroom, is an edible fungus rich in essential nutrients, including proteins (around 30% dry weight), essential amino acids (EAA), dietary fiber, B vitamins, and minerals [4,5]. It also contains numerous bioactive compounds that contribute to its antioxidant and anti-inflammatory properties [5,6]. The optimal temperature for fruiting body growth ranges from 28 °C to 30 °C, with a cultivation-to-harvest period of just 10–15 days [4]. *Volvariella volvacea* is a thermophilic, fast-growing fungus, making it a valuable supplement during the hot season when other edible mushrooms are less viable. It thrives particularly well in high-temperature regions, offering a nutrient-dense food source. Due to its nutritional quality and adaptability, the straw mushroom is widely cultivated and consumed in many countries across Asia, Africa, and Latin America, including China, Thailand, India, Nigeria, and Brazil. Its popularity continues to rise globally as consumers seek plant-based sources of protein and health-promoting foods.

Current research on *Volvariella volvacea* primarily focuses on several key areas. One of the main areas is the optimization of cultivation techniques, including substrate formulations, growing conditions, and management practices [7]. Another significant focus is the extraction and functional exploration of nutritional and bioactive compounds, such as fatty acids [8], polysaccharides [9], and flavonoids [10] and their potential health benefits. Additionally, some studies investigate low-temperature preservation technologies and the autolysis mechanisms of *Volvariella volvacea*, which refer to the degradation or softening of mushrooms during storage [11,12]. The cultivation of *Volvariella volvacea* relies on fiber-rich substrates such as straw, cotton waste, banana leaf waste, bamboo waste, edible mushroom residue, and palm oil empty fruit bunch [13]. Different production regions use various plant-based substrates to optimize cultivation, and variations in carbon-to-nitrogen ratios (C: N = 40–80) often lead to the addition of supplementary substrates like maize power, bran, manure, and fertilizers. These variations contribute to regional and growth-stage variations in nutrient composition [7,13,14,15]. However, research on the authenticity identification of *Volvariella volvacea* based on production region remains limited.

Current technologies for the traceability of agricultural product origins include information traceability [16], chromatographic mass spectrometry [17], spectroscopy [18], DNA analysis [19], and organic component traceability [2]. Among these, the stable isotope technique (SIT) in chromatographic mass spectrometry, primarily applying carbon, nitrogen, hydrogen, and oxygen isotopes (*δ*^13^C, *δ*^15^N, *δ*^2^H and *δ*^18^O), can objectively reflect differences in the production environment and farming practices, which lead to variations in stable isotope fractionation [20]. This enables the precise identification of the authenticity of agricultural product origins and has been applied to the origin identification of edible fungi. For example, Chung et al. [21] employed SIT to trace the geographic origin of shiitake mushrooms cultivated in South Korea (including those grown on both Chinese and South Korean substrates) and China, achieving an origin identification accuracy of 96.4% based on cross-validation. They also applied this technique to trace the origins of *Agaricus bisporus* from South Korea, the Netherlands, and China, achieving 100% accuracy with the optimal support vector machine model [22]. Stable isotopes in edible fungi are primarily influenced by the sources of carbon, nitrogen, and water. For instance, if the carbon source is derived from C3 plants (*δ*^13^C = −35‰ to −20‰), such as straw or waste cotton, the *δ*^13^C value in fungi closely resembles that of C3 plants [20]. If the carbon source is from C4 plants such as maize, sorghum, or sugarcane (*δ*^13^C = −9‰ to −16‰), the *δ*^13^C value is higher [20]. Nitrogen from animal manure with a higher trophic level results in a higher *δ*^15^N value due to the isotopic fractionation, while nitrogen from plant-based or chemical fertilizer results in a lower *δ*^15^N value [21,23]. Water from low-latitude, low-altitude regions near the ocean (known as the “continent effect”) generally leads to higher *δ*^2^H and *δ*^18^O values in fungi [20,24,25]. Additionally, *δ*^2^H and *δ*^18^O values are also influenced by local transpiration and evaporation [20]. Unlike soil-grown crops such as cereals and vegetables, tracing the geographic origin of fungal products requires the comprehensive consideration of both the cultivation substrate and the geographical environment [26]. However, to the best of our knowledge, SIT has not yet been applied to the authenticity identification of *Volvariella volvacea*’s geographic origin.

Therefore, this study aims to evaluate the feasibility of applying SIT for the authenticity of the geographic origin of *Volvariella volvacea*. Through this research, we seek to establish a scientific basis for verifying the authenticity of *Volvariella volvacea*’s origins in the market, thereby providing technical support for origin assurance. Furthermore, the findings will offer a practical tool for regulatory authorities to enhance market supervision and consumer protection, promoting transparency and scientific rigor in the market circulation process.

## 2. Materials and Methods

### 2.1. Sample Collection and Preparation

**Figure 1:**
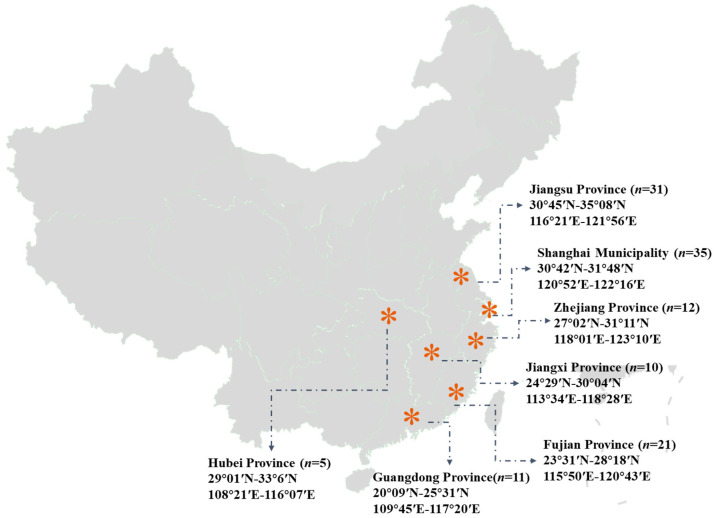
Geographical origin and quantity information of *Volvariella volvacea* samples.

A total of 125 *Volvariella volvacea* samples were collected from local farms and markets across 6 provinces (Fujian, Guangdong, Hubei, Jiangsu, Jiangxi, and Zhejiang) and the municipality of Shanghai in China between May 2023 and May 2024, with detailed information provided in Figure 1. These seven regions are the major production and consumption regions and the focus for authenticity identification in this study. Two samples with unknown origins, collected from the market, were used to validate the predictive performance of the geographic origin authenticity model. After removing impurities, *Volvariella volvacea* specimens with an oval shape and intact cell membranes were selected to eliminate the influence of the growth stage. Fresh samples were homogenized and transferred into sample containers, while dry samples were directly added. The samples were pre-frozen at −18 °C for at least 6 h, freeze-dried at −54 °C for at least 5 days, ground, passed through a 0.15 mm sieve, and stored in an electronic dehumidifier cabinet to prevent water absorption from the local atmosphere.

### 2.2. Protein, Amino Acids and Mineral Contents

The protein, amino acid, and mineral content of 44 selected *Volvariella volvacea* samples were analyzed to evaluate variations in key nutritional components across different geographical origins. The samples included 8 from Fujian, 5 from Guangdong, 5 from Hubei, 8 from Jiangsu, 5 from Jiangxi, 8 from Shanghai, and 7 from Zhejiang. Protein content was measured using the Kjeldahl method (Kjeltec8400, FOSS Analytical, Copenhagen, Denmark), following the Chinese National Standard Method GB 5009.5-2016 [27]. Samples were digested with sulfuric acid and catalysts, converted to ammonium sulfate, and distilled. Ammonia was absorbed by boric acid and titrated with hydrochloric acid, with total nitrogen multiplied by 6.25 to calculate protein content. Amino acid content was analyzed through acid hydrolysis, following the Chinese National Standard Method GB 5009.124-2016 [28]. Samples were hydrolyzed with 6 mol/L HCl, separated by an L-8900 amino acid analyzer (Hitachi Ltd., Tokyo, Japan), and quantified using an external standard method. Mineral element analysis (K, Na, Ca, Mg, Cu, Fe, Mn, Zn, Sr and Se) was performed using microwave digestion (QWave, Questron Technologies Corp., Mississauga, ON, Canada) followed by iCA TQ inductively coupled plasma mass spectrometry (ICP-MS, Thermo Fisher Scientific Inc., Waltham, MA, USA), following the Chinese National Standard Method GB 5009.268-2016 [29]. The sample underwent microwave-assisted digestion to degrade organic matter and release mineral elements, which were then quantified using ICP-MS. Among 18 common amino acids, 7 essential amino acids (EAA) (isoleucine (Ile), leucine (Leu), lysine (Lys), methionine (Met), phenylalanine (Phe), threonine (Thr), and valine (Val)), alanine (Ala), arginine (Arg), aspartic acid (Asp), glutamic acid (Glu), glycine (Gly), histidine (His), proline (Pro) serine (Ser), and tyrosine (Tyr) were quantified, as cysteine and tryptophan were degraded during pretreatment. During acid hydrolysis, glutamine converts to glutamic acid and asparagine to aspartic acid. Thus, the measured glutamic acid represents both the native compound and that derived from glutamine, and similarly for aspartic acid. Each sample was analyzed in triplicate, and the mean value was used as the final result.

### 2.3. Stable Isotope Analysis

The *δ*^13^C, *δ*^15^N, *δ*^2^H, and *δ*^18^O values of *Volvariella volvacea* were determined using a Flash 2000HT elemental analyzer, interfaced to a DELTA V Advantage isotope ratio mass spectrometry system (IRMS, Thermo Fisher Scientific Inc., Bremen, Germany). Instrument parameters were set according to Liu et al. [30]. About 2.40 mg of *Volvariella volvacea* for *δ*^13^C and *δ*^15^N was weighed in triplicate into 5 × 8 mm tin capsules, and 0.20 mg of sample for *δ*^2^H and *δ*^18^O was weighed in triplicate into 4 × 6 mm silver capsules for isotopic analysis. The isotope ratios were determined using the following equation:

*δ*(‰) = [(R_sample_/R_standard_) − 1] × 1000 (1)

where *δ* represents *δ*^13^C, *δ*^15^N, *δ*^2^H or *δ*^18^O; R_sample_ denotes the abundance ratio of the heavy isotope to the light isotope (e.g., ^13^C/^12^C, ^15^N/^14^N, ^18^O/^16^O, ^2^H/^1^H) and R_standard_ is the reference standard isotope ratio. The reference materials used include USGS40 (L-glutamic acid, *δ*^13^C = −26.389 ± 0.042‰, *δ*^15^N = −4.5 ± 0.1‰) and USGS90 (Millet flour, *δ*^13^C = −13.75 ± 0.06‰, *δ*^15^N = 8.84 ± 0.17‰) for *δ*^13^C and *δ*^15^N values, and USGS90 (Millet flour, *δ*^2^H = −13.9 ± 2.4‰, *δ*^18^O = 35.90 ± 0.29‰) and USGS91 (Rice flour, *δ*^2^H = −45.7 ± 7.4‰, *δ*^18^O = 21.13 ± 0.44‰) for *δ*^2^H and *δ*^18^O values. A sample of *Volvariella volvacea* was chosen as a quality control measure and included as a working standard after every 10 unknown samples. The instrumental precision was lower than ±0.1‰ for *δ*^13^C, ±0.2‰ for *δ*^15^N, ±2.0‰ for *δ*^2^H and ±0.5‰ for *δ*^18^O, respectively.

### 2.4. Statistical Analysis and Chemometric Methods

One-way analysis of variance (ANOVA) and multiple comparisons analysis were conducted to evaluate the differences in protein, amino acid, mineral content, and *δ*^13^C, *δ*^15^N, *δ*^2^H, and *δ*^18^O values among *Volvariella volvacea* from different geographic origins using Matlab R2020a software. Descriptive analysis was performed using violin plots and scatter plots, where each off-diagonal cell displays the relationship between pairs of isotopes, and the diagonal cells present the histograms for each isotope. To accurately identify the geographic origin of *Volvariella volvacea* samples from the major production and consumption regions, principal component analysis (PCA) and partial least squares discriminant analysis (PLS-DA) were applied using SIMCA 14.1 software (Umetrics, Umeå, Sweden) [31]. Before applying PCA and PLS-DA analyses, data were first mean-centered and scaled to unit variance. PCA, utilizing singular value decomposition, was employed to extract principal components (PCs) and generate the score and loading plots to elucidate sample clustering and variable contributions, with model performance evaluated by R²X and Q² via 4-fold cross-validation [31]. Conversely, PLS-DA maximized the covariance between predictor and response variables by iteratively extracting latent variables to construct a linear discriminant model, classify samples based on Mahalanobis distances, and identify key variables using the variable importance in projection (VIP), with model parameters (R²X, R²Y, and Q²) assessed through 7-fold cross-validation [31]. The model’s classification ability is reflected by its accuracy.

## 3. Results and Discussion

### 3.1. Geographical Variation in Nutritional Composition of Volvariella volvacea

**Figure 2:**
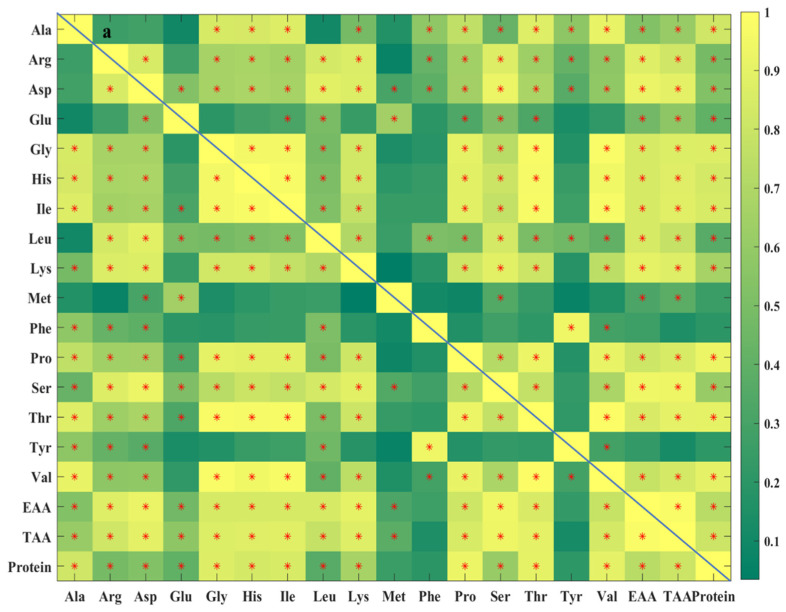

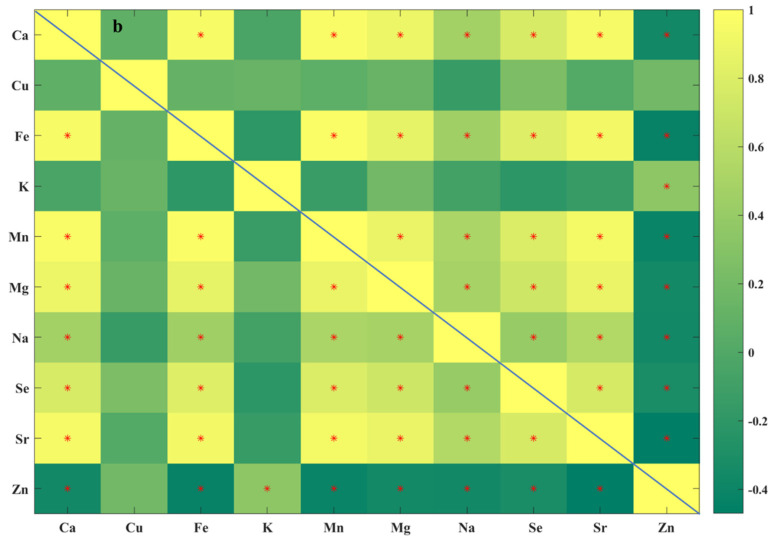
Pearson correlation analysis of amino acids, total essential amino acid (EAA), total amino acid (TAA) and protein content (**a**), and mineral elements (**b**) of *Volvariella volvacea*. “*” denotes significant correlations (*p* < 0.05).

The mean protein content of the 45 selected *Volvariella volvacea* samples was 31.30 ± 2.67 g/100 g. Guangdong samples (33.46 ± 3.12 g/100 g, Table 1) exhibited significantly higher protein content than those from Fujian, Hubei, and Zhejiang (*p* < 0.05). These results indicate that the protein content of *Volvariella volvacea* is significantly higher than that of common cereals (approximately 10%) [32], suggesting its potential as a high-quality protein source in human nutrition. Furthermore, Guangdong samples may provide even greater nutritional value. This finding is consistent with previous reports on the protein content of *Volvariella volvacea* by Ahlawat et al. [4] and Zakhary et al. [33], as well as other edible mushrooms reported by Beluhan et al. [34] and Assemie et al. [35]. Among the 16 amino acids in *Volvariella volvacea* samples from seven major production areas, Glu had the highest content (3.78 ± 0.46 g/100 g), while Leu had the highest content (1.81 ± 0.2 g/100 g) among the EAA, similar to the amino acid composition reported in other edible mushrooms [35]. Additionally, most amino acids showed significant regional variations in content (Table 1). For instance, Fujian samples exhibited significantly higher Phe and Tyr content, while Ala, Arg, Glu, Gly, His, Ile, Lys, Pro, Ser, Thr, and Val were significantly lower (*p* < 0.05). In contrast, Guangdong samples had significantly higher Ala, Gly, His, Ile, and Val content, whereas Arg, Glu, Lys, Phe, and Tyr levels were significantly lower. In Hubei, Met and Thr content were significantly higher, whereas Glu, Gly, Lys, Ser, Thr, and Val content were significantly lower. Jiangsu samples exhibited significantly higher Glu and Met content, with significantly lower Phe content. In Jiangxi, Phe and Tyr content were significantly higher. Shanghai samples showed significantly higher Ala, Arg, Gly, Ile, Pro, Ser, Thr, Tyr, and Val content, while Glu and Met contents were significantly lower. Zhejiang samples exhibited significantly higher Tyr content, but significantly lower Arg, Gly, His, Ile, Lys, Pro, Thr, and Val. Therefore, based on the differences in amino acid contents among *Volvariella volvacea* samples from different regions, specific production areas can be considered for targeted amino acid supplementation. These findings also demonstrate that the amino acid composition of *Volvariella volvacea* is distinctive to the region of origin. Moreover, these differences may be attributed to variations in cultivation substrates, harvest time, and genetic factors, all of which influence amino acid synthesis and metabolism in *Volvariella volvacea* [36]. Although significant regional differences were observed in specific amino acids, there were no significant differences in Leu, total EAA or TAA content. This could be due to the compensatory relationship among the different amino acids, which helps maintain the stability of TAA content. For example, in each production area, some amino acids exhibit significantly higher concentrations, while others show significantly lower levels. Additionally, certain amino acids exhibit strong correlations with one another. For instance, Guangdong and Shanghai samples both showed significantly higher Gly, Ile, and Val content, while these three amino acids were significantly lower in the Fujian and Zhejiang samples. As shown in Figure 2a, the correlation coefficients (r) between Gly, Ile, and Val were as follows: r_(Gly-Ile)_ = 0.96, r_(Gly-Val)_ = 0.98, and r_(Ile-Val)_ = 0.97. Many other amino acids also exhibited statistically significant positive correlations. The content of individual EAA in this study was found to be higher than that reported by Ahlawat et al. [4], possibly due to differences in mushroom variety, cultivation substrates, and environmental conditions. Notably, Glu had the highest content among the measured amino acids, consistent with the amino acid distribution reported by Beluhan et al. in wild edible mushrooms from Croatia [34]. This suggests that different varieties of edible mushrooms share certain similarities in their amino acid composition.

Significant differences were observed in the content of mineral elements in *Volvariella volvacea* samples from seven major production areas (Table 1). For example, Fujian samples exhibited significantly higher contents of Ca, Fe, Mg, Na, Se, and Sr, while Zn content was significantly lower (*p* < 0.05). Guangdong samples had significantly lower levels of Fe, Mn, Na, Se, and Zn. The samples from Hubei showed significantly higher levels of Ca, Fe, Mn, Mg, Se, and Sr, with significantly lower Zn content. Jiangsu samples exhibited significantly higher Zn content. Jiangxi samples had significantly lower Fe and Mg contents. Shanghai samples had significantly lower contents of Ca, Fe, Mn, Mg, Na, Sr, and Zn. Zhejiang samples exhibited significantly higher contents of Ca, Fe, Mn, Mg, Se, and Sr, with significantly lower Zn content. These results indicate that each region exhibits distinct mineral element distribution characteristics. Fujian, Hubei and Zhejiang samples may serve as good dietary sources of Ca, Fe, Mn, Mg, Se, and Sr, while Jiangsu samples may be rich in dietary zinc. Additionally, considerable variations were observed not only between different production areas but also within the same area. For instance, the Ca and Fe contents in the Hubei samples were 1203.11 ± 466.56 mg/kg and 770.71 ± 159.32 mg/kg, respectively, while in the Shanghai samples, these values were only 221.07 ± 121.26 mg/kg and 58.85 ± 16.84 mg/kg, with the standard deviations for Ca and Fe in the Hubei samples being quite large. These differences may be influenced by various factors, including the cultivation substrates, the varieties of *Volvariella volvacea*, and environmental factors such as climate and water quality, highlighting the regional characteristics in the accumulation of mineral elements. There were also notable correlations between the mineral elements in *Volvariella volvacea*. For instance, Ca, Fe, Mn, Mg, and Sr were found to be high in Fujian, Hubei, and Zhejiang samples but significantly lower in Shanghai samples. As shown in Figure 2b, these mineral elements exhibit significant strong positive correlations, with the following correlation coefficients: r_(Ca-Fe)_ = 0.96, r_(Ca-Mn)_ = 0.97, r_(Ca-Mg)_ = 0.90, r_(Ca-Sr)_ = 0.94, r_(Fe-Mn)_ = 0.98, r_(Fe-Mg)_ = 0.86, r_(Fe-Sr)_ = 0.93, r_(Mn-Mg)_ = 0.88, r_(Mn-Sr)_ = 0.94, and r_(Mg-Sr)_ = 0.88. The mineral element in *Volvariella volvacea* was higher than that reported by Ramkumar et al. [6] and Okoro et al. [37], which may also be attributed to differences in *Volvariella volvacea* varieties, cultivation substrates, and growing environments. Furthermore, the Ca content was higher than that of oyster mushrooms, while the contents of K, Na, Zn, and Mn were lower. The contents of Ca, Fe, K, Mn, Na, and Zn were similar to those found in *Pleurotus eryngii* [38]. These results indicate that *Volvariella volvacea*, like other edible mushrooms, is rich in mineral elements, with certain minerals being higher due to cultivation conditions.

Overall, the protein, amino acid, and mineral profiles of *Volvariella volvacea* vary according to geographic origin, indicating its potential as a dietary supplement for specific amino acids and minerals that may be deficient in certain diets. Future studies can increase the sample size from each region to help reduce internal variation and further validate the differences in amino acid and mineral content. These results underscore the importance of ensuring the authenticity of the geographic origin of *Volvariella volvacea*, as its nutritional composition exhibits regional characteristics. Therefore, SIT was applied to trace the origin of *Volvariella volvacea*.

### 3.2. Stable Isotope Distribution of Volvariella volvacea from Different Origins

**Figure 3:**
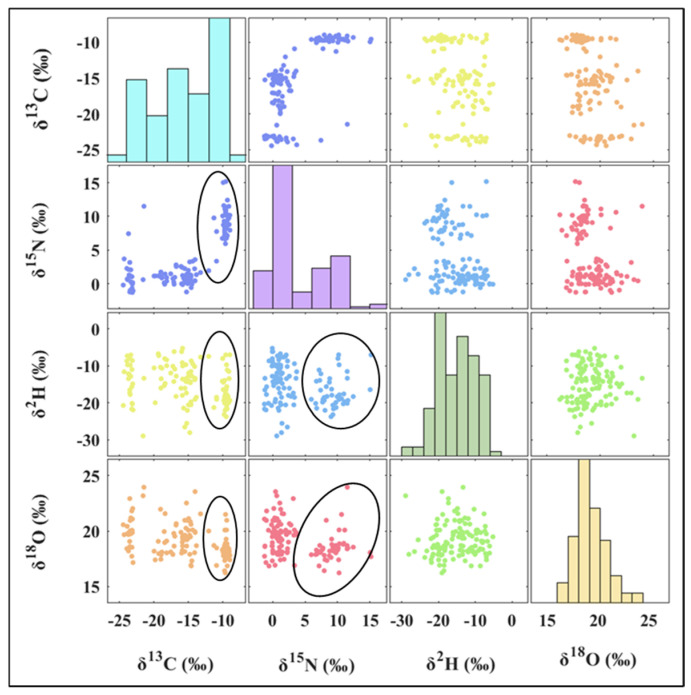
Scatter plot of stable isotope distribution and isotope relationships in *Volvariella volvacea*, where black ellipses indicate production regions with significantly higher δ^13^C and δ^15^N values.

**Figure 4:**
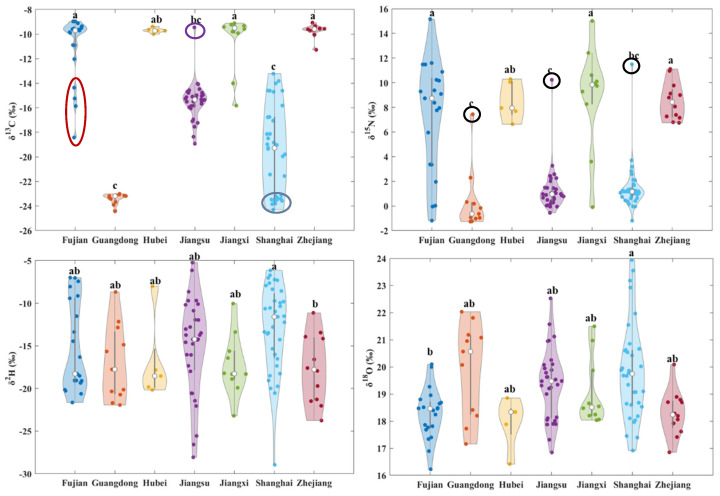
Stable isotope distributions of *Volvariella volvacea* from different geographical origins, where the white circle represents the median. The samples marked with circles represent outliers or values requiring special explanation. Different letters above a violin indicate a significant difference for each cultivation method (*p* < 0.05).

Stable isotopes also exhibited variations across different origins (Figure 3 and Figure 4, Table 2). The *δ*^13^C values of samples from known origins ranged from −24.43‰ to −8.97‰, with a mean value of −15.36‰ ± 5.08‰. The highest frequency distribution was observed between −9‰ and −10‰, as shown in the bar chart in Figure 3 (light turquoise). The range, standard deviation, and mean value were notably broader and higher compared to those reported for shiitake mushrooms (around −25‰) [26] and *Agaricus bisporus* (around −24‰) [22]. These findings suggest that the carbon sources used in *Volvariella volvacea* cultivation may be more diverse, encompassing both C3 plants and C4 plants. For example, *Volvariella volvacea* samples from Guangdong showed an average *δ*^13^C value of −23.41‰ ± 0.43‰, indicating that the cultivation substrate may be C3-based materials such as rice straw and/or cotton waste. In contrast, samples from regions such as Hubei (−9.70‰ ± 0.22‰), Jiangxi (−10.57‰ ± 2.35‰), and Zhejiang (−9.72‰ ± 0.54‰) may mainly utilize C4 plant-based materials like maize, sorghum, or sugarcane waste. Furthermore, regions like Fujian (−10.98‰ ± 2.67‰), Jiangsu (−15.46‰ ± 1.61‰), and Shanghai (−19.48‰ ± 3.61‰) may employ a combination of C3 and C4 plants. In these areas, some farms use either C3 (indicated by blue circle in Figure 4) or C4 plants (indicated by a purple circle in Figure 4) individually. These results suggest that variations in the cultivation substrates may occur even within the same geographic origin and may pose a challenge for SIT to accurately identify *Volvariella volvacea* from these production areas. As a result, *Volvariella volvacea* from different origins exhibited variations in *δ*^13^C values. The *δ*^13^C values from Fujian, Hubei, Jiangxi, and Zhejiang differed significantly from those of Guangdong and Shanghai (*p* < 0.05). Additionally, Fujian, Jiangxi, and Zhejiang samples also showed significant differences compared to those from Jiangsu (*p* < 0.05).

The *δ*^15^N values of *Volvariella volvacea* samples ranged from −1.30‰ to 15.16‰, with a mean of 3.88‰ ± 4.33‰ (Figure 3, light purple bar chart). The highest frequency distribution occurred between 0‰ and 2‰. This is slightly higher than the reported mean *δ*^15^N value for shiitake mushrooms (−3.08‰) [26] but lower than that for *Agaricus bisporus* (11.31‰) [22]. This difference may be attributed to the varying nitrogen sources in the cultivation substrates. Generally, different nitrogen sources display distinct *δ*^15^N values, with animal manure (*δ*^15^N = 8.1‰ ± 3.9‰) and plant-based fermented fertilizers (*δ*^15^N ≥ 2‰) yielding higher *δ*^15^N values compared to synthetic chemical fertilizers (*δ*^15^N = −0.2‰ ± 2.1‰) [20,23]. Shiitake mushrooms typically rely on plant-based nitrogen sources such as sawdust, whereas *Agaricus bisporus* cultivation often includes animal manure [22,26]. Therefore, it can be inferred that approximately 34.40% of *Volvariella volvacea* samples may have been cultivated on substrates containing animal manure, including 71.43% of the samples from Fujian, 100% from Hubei, 80% from Jiangxi, and 100% from Zhejiang. Additionally, Jiangsu (*n* = 1, black circle in Figure 4), Shanghai (*n* = 1, black circle in Figure 4), Guangdong (*n* = 1, black circle in Figure 4), and two unknown origin samples (10.41‰ and 9.51‰) also exhibited elevated *δ*^15^N values. The differences in nitrogen source nutrient levels in the cultivation substrates resulted in significant variations in the *δ*^15^N values of *Volvariella volvacea* from Fujian, Jiangxi, and Zhejiang compared to those from Guangdong, Jiangsu, and Shanghai (*p* < 0.05). Furthermore, the samples from Hubei showed significant differences compared to those from Guangdong and Jiangsu (*p* < 0.05).

The *δ*^2^H values of *Volvariella volvacea* samples from all regions ranged from −28.96‰ to −5.24‰, including two unknown origins (−19.24‰ and −21.47‰), with a mean value of −15.15‰ ± 5.19‰ (Figure 3, light green bar chart). The distribution of *δ*^2^H values was relatively uniform across this range. Only the *δ*^2^H values of *Volvariella volvacea* from Shanghai were significantly higher than those from Zhejiang (*p* < 0.05), despite Shanghai being adjacent to Zhejiang and having a slightly higher latitude. This may be attributed to Shanghai’s generally low altitude and closer proximity to the ocean, as well as the fact that *Volvariella volvacea* is primarily cultivated in greenhouses. Since it is a thermophilic species, the temperature and humidity within the greenhouse may have a stronger influence on evaporation and transpiration.

The *δ*^18^O values of *Volvariella volvacea* exhibited an approximately normal distribution, ranging from 16.22‰ to 23.95‰, with 32.00% of the samples falling between 18‰ and 19‰, with a mean of 19.18‰ ± 1.51‰. The *δ*^18^O values of the two unknown samples (18.07‰ and 18.41‰) also fell within this range. Significant differences in *δ*^18^O values were observed only between Shanghai and Fujian. Despite Fujian being at a lower latitude, its samples did not exhibit higher *δ*^18^O values than those from Shanghai. The differences are more likely influenced by variations in altitude, proximity to the ocean, and the evaporative transpiration effects of the local microclimate. As observed by Chung et al. [26] in their stable isotope analysis of the geographic origin differences in South Korean shiitake mushrooms, the *δ*^18^O values in high-latitude regions, such as Cheongyang shiitake (24.59‰), were higher than those in the low-latitude regions like Gurye shiitake (22.89‰), which does not align with the expected latitude effect. Similarly, the *δ*^2^H and *δ*^18^O values in *Volvariella volvacea* did not show the same positive proportional trend typically observed in atmospheric precipitation (*δ*^2^H = 8*δ*^18^O + 10) [24,25], as shown in the scatter plot of the green dots in Figure 3.

The results indicate that the *δ*^13^C, *δ*^15^N, *δ*^2^H, and *δ*^18^O values of *Volvariella volvacea* from different production areas are influenced by a combination of factors, including the carbon and nitrogen sources in the cultivation substrate, water sources, and local microclimates. As studied by Chung et al. [26] and Suzuki et al. [39], the carbon stable isotopes in shiitake mushrooms are influenced by the type of cultivation substrate, such as wood log or mycelial cultivation, and the nitrogen stable isotopes are determined by the characteristics of nitrogen-based nutrient sources (e.g., intensity, type, availability) in the cultivation medium. Based on the distribution differences of stable isotopes, particularly carbon and nitrogen from different origins, and the relationships between pairs of stable isotopes, *Volvariella volvacea* from the seven major production areas can be preliminarily classified into two groups: Fujian, Hubei, Jiangxi, and Zhejiang as one group, characterized by significantly higher *δ*^13^C and *δ*^15^N values (Figure 3, black ellipses), and Guangdong, Jiangsu, and Shanghai as another. Within each group, stable isotope values overlap significantly, making it difficult to precisely distinguish the origins. PCA and PLS-DA methods can integrate the differences in individual stable isotopes from different regions, potentially providing a new perspective for the identification of *Volvariella volvacea* origins.

### 3.3. Origin Discrimination of Volvariella volvacea

**Figure 5:**
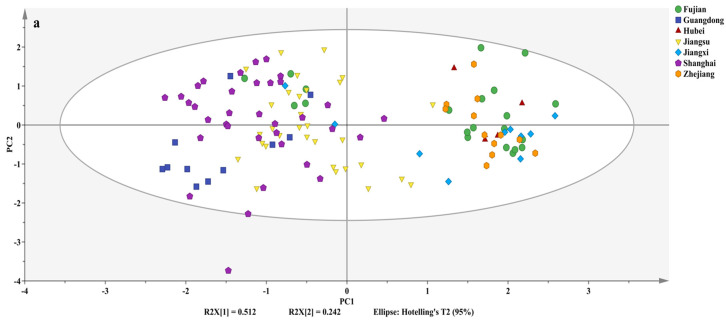

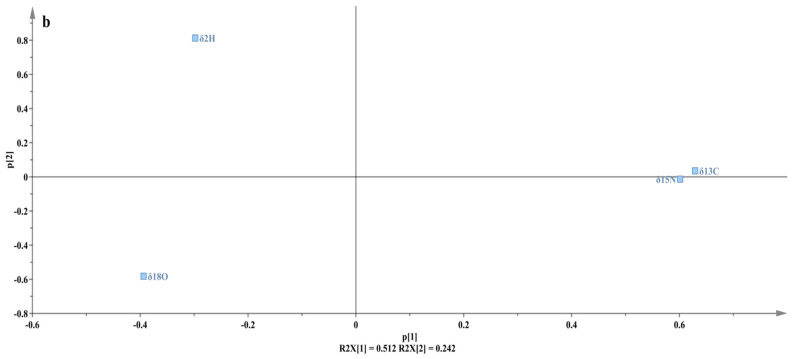
Plot of PCA scores (**a**) and loadings plot (**b**) of *Volvariella volvacea* from different geographical origins.

PCA can simplify the data structure and reduce redundant information by extracting the most informative features from multi-dimensional data, thereby improving the effectiveness and efficiency of agricultural product origin discrimination. The first two principal components (PC1 and PC2) explain 2.048 and 0.968 of the total variation in the original data, accounting for 51.20% (R^2^X[1]) and 24.20% (R^2^X[2]) of the variance, respectively (Figure 5a). They contributed to a cumulative predictive ability (Q²cum) of 0.124, showing the distribution of *Volvariella volvacea* samples from different regions. Samples from Guangdong, Jiangsu, and Shanghai, along with five samples from Fujian and two from Jiangxi, were primarily located on the left side of the vertical axis, while those from Fujian, Hubei, Jiangxi, Zhejiang, two from Shanghai and five from Jiangsu were mainly on the right side. This distribution is likely attributed to variations in the proportions of C3 and C4 plants used in the cultivation substrates, as well as the use of high-nutrient manure, as indicated in Figure 4. In addition, as shown in Figure 5b, the loading plot displayed that the variables with the greatest influence on the first principal component (PC1) were the *δ*^13^C and *δ*^15^N values. PCA divided the samples into two major groups: one consisting of Guangdong, Jiangsu, and Shanghai, and the other consisting of Fujian, Hubei, Jiangxi, and Zhejiang. Shanghai samples overlapped with those of Guangdong and Jiangsu, while Guangdong and Jiangsu were relatively distinct. There was significant overlap among samples from Fujian, Hubei, Jiangxi, and Zhejiang, making it difficult to distinguish between them.

The clustering differences among *Volvariella volvacea* origins were closely related to the distribution differences in their *δ*^13^C, *δ*^15^N, *δ*^2^H, and *δ*^18^O values (Figure 3). Origins with similar stable isotope ratios tended to cluster together, while those with larger differences were more separated. Although PCA transformed the data into a set of linearly independent new variables, the main production regions of *Volvariella volvacea* could still be classified into two major categories, with subgroups within each category showing clustering patterns. However, there was some overlap between the samples from Jiangsu and Shanghai and those from Fujian, Hubei, Jiangxi, and Zhejiang. Therefore, the supervised algorithm PLS-DA was used to improve the identification accuracy of *Volvariella volvacea* from different origins.

**Figure 6:**
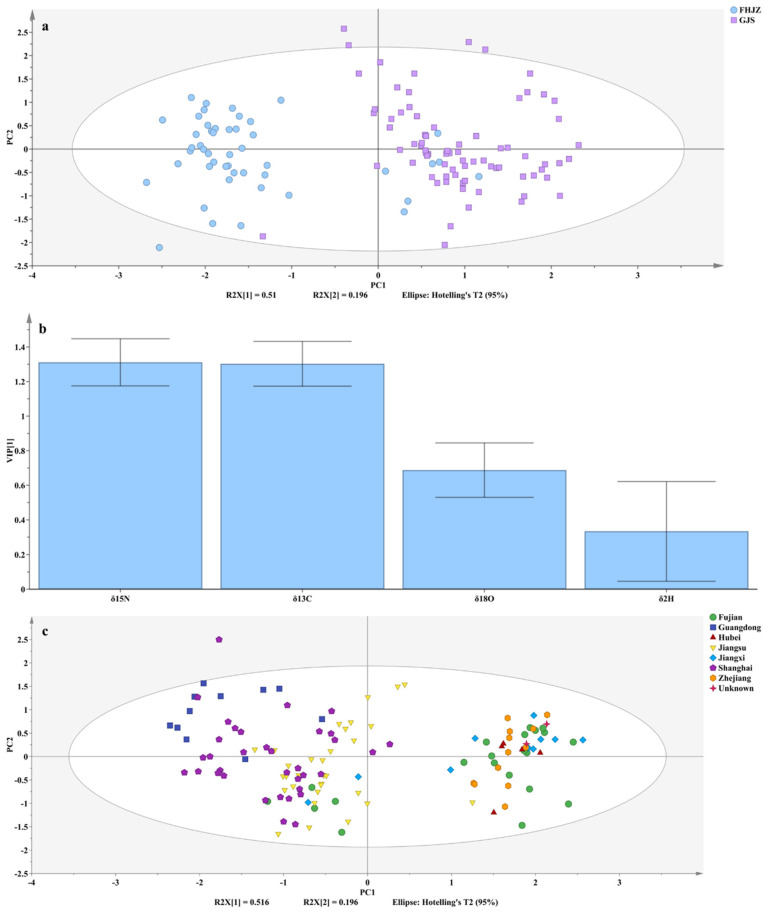
Plot of PLS-DA scores (**a**); plot of variable importance in projection (**b**) of *Volvariella volvacea* from the FHJZ (Fujian, Hubei, Jiangxi, Zhejiang) group and the GJS (Guangdong, Jiangsu, Shanghai) group; and plot of PLS-DA scores (**c**) for samples from seven major producing regions and unknown regions.

The precise identification of the two main production regions was achieved using a PLS-DA method, which establishes a discriminant model by extracting the principal components that most effectively distinguish between different classes in the feature space [31]. This method takes into account not only the variance within the input data but also the differences between the classes. The identification accuracy for the Fujian, Hubei, Jiangxi and Zhejiang (FHJZ) group samples was 85.42%, with two Jiangxi and five Fujian samples misclassified (Figure 6a), likely due to their lower *δ*^13^C and *δ*^15^N values (Figure 4). For the Guangdong, Jiangsu and Shanghai (GJS) group, the accuracy was 98.70%, with only one Jiangsu sample misclassified (Figure 6a). This was primarily attributed to the higher *δ*^13^C and *δ*^15^N values of this sample compared to other Jiangsu samples (Figure 4). Furthermore, the order of VIP was *δ*^15^N > *δ*^13^C > 1 > *δ*^18^O > *δ*^2^H, indicating that the *δ*^15^N and *δ*^13^C values were the most influential variables in the PLS-DA model (Figure 6b). This differs from the study by Chung et al. [26], which used SIT to identify shiitake mushrooms from China and South Korea. In that study, *δ*^15^N and *δ*^18^O values were key variables in the orthogonal partial least squares discriminant analysis model, likely due to differences in nitrogen sources, latitude, altitude, and proximity to the ocean between the two countries. This PLS-DA model selected two PCs, explaining 70.60% of the variation in the stable isotopes (R²Xcum) by capturing 2.04 and 0.784 of the total variation in the original data. It demonstrated an R²Ycum of 0.702 in distinguishing the two major origin groups of *Volvariella volvacea* and a Q²cum of 0.685 for predictive ability. Two unknown samples were used to validate the PLS-DA model. When classified into the FHJZ group, the overall accuracy of the model increased to 93.70%, indicating its strong predictive performance. These results indicate that SIT can effectively distinguish between different regions of *Volvariella volvacea*, even when the regions likely use similar cultivation substrates.

The regions within each major group of *Volvariella volvacea* were further distinguished pairwise using the PLS-DA model (Figure 6c and Table 3). In the GJS group, the classification accuracy for Guangdong and Jiangsu samples was 100%, and it was 86.96% for Guangdong and Shanghai and 81.82% for Jiangsu and Shanghai. As shown in Figure 6c, some Shanghai samples overlapped with those from Guangdong and Jiangsu. According to the VIP ranking, the *δ*^13^C value, primarily influenced by the carbon sources of the cultivation substrate, was the most important variable in this group, as supported by its distribution in Figure 4. The primary carbon sources for Guangdong samples were likely C3 plants (e.g., straw and cotton waste), while Jiangsu samples were mainly derived from C4 plants (e.g., maize-related products) or mixtures of C3 and C4 plants. In contrast, Shanghai samples were sourced from either mixed C3 and C4 plants or predominantly C3 plants. Additionally, the *δ*^2^H value played a critical role in distinguishing Guangdong and Shanghai samples, likely due to geographical factors. Guangdong sampling sites were farther inland despite the lower latitude, while Shanghai was closer to the coast. In the FHJZ group, Fujian samples achieved identification accuracy rates of 100%, 100%, and 85.71% in discrimination models against Hubei, Jiangxi, and Zhejiang samples, respectively, indicating distinct regional features. In the discrimination models between Hubei and Jiangxi/Zhejiang samples, the Jiangxi and Zhejiang samples demonstrated high classification accuracy rates of 100% and 91.67%, respectively. Notably, the mutual discrimination accuracy between Jiangxi and Zhejiang samples was relatively low. Moreover, the two unknown samples in this group could not be precisely classified into a specific region (Figure 6c). This phenomenon may be attributed to the following factors: (1) the cultivation substrates in all four regions were predominantly composed of C4 plants, resulting in similar substrate compositions; (2) the relatively limited sample sizes from Hubei (*n* = 5), Jiangxi (*n* = 10), and Zhejiang (*n* = 12). VIP analysis revealed that while the *δ*^13^C value was the key variable in Fujian–Hubei and Fujian–Zhejiang discrimination models, the *δ*^18^O emerged as the most important variable in other models. This discrepancy may be associated with the presence of C3/C4 mixed cultivation substrates in Fujian (indicated by the red ellipse in Figure 3) and could also be influenced by a combination of factors, including regional latitude, distance from the sea, and microclimate conditions within cultivation facilities.

Our findings are similar to those of previous studies [21,22,26], indicating that SIT, combined with supervised discriminant analysis methods, can clearly discriminate various mushrooms according to their geographical origin, whether across countries, continents, or regions within the same country. However, due to the combined effects of cultivation substrate types, geographical information, cultivation facilities, and sample sizes, our PLS-DA model could not provide perfect regional discrimination for the Hubei, Jiangxi, and Zhejiang samples within the FHJZ group. This limitation is consistent with the findings of Chung et al. [40], who reported that their PLS-DA model, based on stable isotopes, failed to effectively discriminate *Agaricus bisporus* from Boryung, Buyeo, and Daegu regions in South Korea. To improve the accuracy of SIT in determining the geographical origin of *Volvariella volvacea* and detecting mislabeling, future studies should focus on (1) increasing sample sizes from different geographical origins; (2) analyzing stable isotope profiles of cultivation substrates; and (3) incorporating strain-specific genetic data. Furthermore, integrating stable isotope analysis with these parameters offers a robust framework for authenticating mushroom origins across the production chain, effectively addressing food safety and quality assurance concerns, and combating mislabeling and adulteration practices.

## 4. Conclusions

This study revealed that the main nutritional components of *Volvariella volvacea*, including protein, common amino acids, and mineral elements, varied according to geographic origin, exhibiting distinct regional characteristics, emphasizing the importance of ensuring the authenticity of the geographic origin of *Volvariella volvacea*.

SIT was employed to verify the geographic origin of samples from seven major production area. Differences in the cultivation substrates led to higher *δ*^13^C and *δ*^15^N values in the Fujian, Hubei, Jiangxi, and Zhejiang samples. Additionally, the latitude effect, continental effect, and local microclimate evaporation resulted in a significantly higher *δ*^2^H value in Shanghai compared to Zhejiang and a higher *δ*^18^O value in Shanghai compared to Fujian. Based on the distribution differences, the seven major production areas could be initially divided into two main groups—Fujian, Hubei, Jiangxi, and Zhejiang, and Guangdong, Jiangsu, and Shanghai—thereby ensuring the authenticity of *Volvariella volvacea* between these two groups. PCA largely confirmed this division. The PLS-DA model achieved a 93.60% accuracy for the two groups, with the *δ*^15^N and *δ*^13^C values as the key variables. It correctly classified more than 80% of pairwise combinations within Guangdong, Jiangsu, and Shanghai samples, while in the Fujian, Hubei, Jiangxi, and Zhejiang group, only Fujian–Hubei and Hubei–Jiangxi exceeded 80% accuracy. Other combinations still need improvement. These results demonstrate the feasibility of using SIT for tracing the geographic origin of *Volvariella volvacea*. However, further research is needed to increase sample sizes, diversify cultivation substrates, and improve model accuracy, robustness, and predictive performance. This will provide valuable technical support for market regulation and transparency in the *Volvariella volvacea* supply chain.

## Figures and Tables

**Table 1:** Nutritional composition distribution of *Volvariella volvacea*.

Nutrients	Fujian	Guangdong	Hubei	Jiangsu	Jiangxi	Shanghai	Zhejiang
Protein	29.77 ± 1.59 ^b^	33.46 ± 3.12 ^a^	29.56 ± 1.11 ^b^	32.42 ± 1.06 ^ab^	31.78 ± 3.92 ^ab^	32.32 ± 1.80 ^ab^	29.49 ± 1.40 ^b^
Ala	1.47 ± 0.14 ^b^	2.50 ± 0.50 ^a^	1.54 ± 0.13 ^ab^	1.83 ± 0.12 ^ab^	1.61 ± 0.13 ^ab^	1.90 ± 0.17 ^a^	1.51 ± 0.08 ^ab^
Arg	1.25 ± 0.10 ^b^	1.23 ± 0.17 ^b^	1.29 ± 0.10 ^ab^	1.31 ± 0.50 ^ab^	1.33 ± 0.10 ^ab^	1.48 ± 0.24 ^a^	1.15 ± 0.09 ^b^
Asp	2.18 ± 0.17 ^a^	2.21 ± 0.20 ^a^	2.24 ± 0.13 ^a^	2.34 ± 0.39 ^a^	2.43 ± 0.22 ^a^	2.47 ± 0.31 ^a^	2.20 ± 0.14 ^a^
Glu	3.69 ± 0.31 ^b^	3.71 ± 0.38 ^b^	3.77 ± 0.18 ^b^	4.61 ± 0.28 ^a^	4.04 ± 0.54 ^ab^	3.62 ± 0.61 ^b^	3.96 ± 0.43 ^ab^
Gly	1.01 ± 0.07 ^b^	1.19 ± 0.15 ^a^	0.98 ± 0.05 ^b^	1.11 ± 0.11 ^ab^	1.08 ± 0.06 ^ab^	1.19 ± 0.08 ^a^	0.99 ± 0.06 ^b^
His	0.53 ± 0.04 ^b^	0.67 ± 0.09 ^a^	0.59 ± 0.05 ^ab^	0.62 ± 0.07 ^ab^	0.57 ± 0.04 ^ab^	0.65 ± 0.08 ^a^	0.52 ± 0.03 ^b^
Ile	1.14 ± 0.08 ^b^	1.40 ± 0.19 ^a^	1.19 ± 0.10 ^ab^	1.27 ± 0.15 ^ab^	1.22 ± 0.09 ^ab^	1.32 ± 0.13 ^a^	1.12 ± 0.06 ^b^
Leu	1.77 ± 0.14 ^a^	1.71 ± 0.26 ^a^	1.83 ± 0.12 ^a^	1.89 ± 0.26 ^a^	1.95 ± 0.17 ^a^	1.99 ± 0.41 ^a^	1.74 ± 0.09 ^a^
Lys	1.46 ± 0.11 ^b^	1.59 ± 0.16 ^b^	1.55 ± 0.10 ^b^	1.53 ± 0.20 ^ab^	1.70 ± 0.20 ^ab^	1.82 ± 0.18 ^a^	1.45 ± 0.06 ^b^
Met	0.78 ± 0.07 ^ab^	0.83 ± 0.10 ^ab^	0.88 ± 0.03 ^a^	0.85 ± 0.05 ^a^	0.79 ± 0.06 ^ab^	0.74 ± 0.10 ^b^	0.87 ± 0.10 ^ab^
Phe	1.15 ± 0.09 ^a^	0.81 ± 0.09 ^c^	1.09 ± 0.08 ^ab^	0.86 ± 0.09 ^bc^	1.27 ± 0.13 ^a^	1.08 ± 0.18 ^ab^	1.13 ± 0.06 ^ab^
Pro	1.21 ± 0.07 ^bc^	1.35 ± 0.13 ^ab^	1.19 ± 0.06 ^bc^	1.35 ± 0.08 ^ab^	1.28 ± 0.15 ^abc^	1.37 ± 0.07 ^a^	1.17 ± 0.03 ^c^
Ser	1.18 ± 0.08 ^b^	1.26 ± 0.11 ^ab^	1.17 ± 0.05 ^b^	1.28 ± 0.12 ^ab^	1.30 ± 0.10 ^ab^	1.34 ± 0.19 ^a^	1.19 ± 0.06 ^ab^
Thr	1.23 ± 0.08 ^b^	1.46 ± 0.17 ^ab^	1.20 ± 0.09 ^b^	1.36 ± 0.10 ^ab^	1.33 ± 0.11 ^ab^	1.42 ± 0.09 ^a^	1.23 ± 0.06 ^b^
Tyr	0.80 ± 0.09 ^a^	0.54 ± 0.07 ^b^	0.74 ± 0.08 ^a^	0.65 ± 0.07 ^ab^	0.91 ± 0.07 ^a^	0.78 ± 0.13 ^a^	0.79 ± 0.09 ^a^
Val	1.29 ± 0.11 ^b^	1.60 ± 0.22 ^a^	1.30 ± 0.06 ^b^	1.43 ± 0.16 ^ab^	1.39 ± 0.11 ^ab^	1.52 ± 0.09 ^a^	1.26 ± 0.07 ^b^
EAA	8.81 ± 0.63 ^a^	9.40 ± 0.98 ^a^	9.04 ± 0.31 ^a^	9.19 ± 0.79 ^a^	9.65 ± 0.84 ^a^	9.89 ± 1.05 ^a^	8.80 ± 0.48 ^a^
TAA	22.14 ± 1.55 ^a^	24.07 ± 2.56 ^a^	22.55 ± 1.23 ^a^	24.19 ± 1.57 ^a^	24.21 ± 2.26 ^a^	24.72 ± 2.42 ^a^	22.29 ± 1.37 ^a^
Ca	985.01 ± 465.73 ^a^	421.69 ± 341.62 ^ab^	1203.11 ± 466.56 ^a^	706.93 ± 517.23 ^ab^	723.17 ± 587.96 ^ab^	221.07 ± 121.26 ^b^	1110.72 ± 239.681 ^a^
Cu	46.55 ± 9.06 ^a^	36.29 ± 16.53 ^a^	38.42 ± 10.61 ^a^	42.96 ± 14.41 ^a^	42.11 ± 11.33 ^a^	44.77 ± 22.39 ^a^	47.72 ± 5.24 ^a^
Fe	484.42 ± 184.85 ^ab^	127.87 ± 102.97 ^cd^	770.71 ± 159.32 ^a^	281.90 ± 145.59 ^bc^	361.62 ± 106.75 ^b^	58.85 ± 16.84 ^d^	652.45 ± 134.24 ^a^
K	42.72 ± 3.06 ^a^	43.64 ± 2.15 ^a^	42.87 ± 1.04 ^a^	45.77 ± 5.04 ^a^	40.30 ± 0.80 ^a^	41.20 ± 3.13 ^a^	41.48 ± 2.01 ^a^
Mn	24.95 ± 9.33 ^ab^	15.85 ± 6.02 ^b^	25.48 ± 2.37 ^a^	17.99 ± 4.74 ^ab^	22.34 ± 10.88 ^ab^	12.26 ± 1.89 ^b^	26.86 ± 3.32 ^a^
Mg	1242.25 ± 124.71 ^ab^	1121.81 ± 73.92 ^bc^	1338.93 ± 61.17 ^a^	1120.69 ± 165.87 ^abc^	1076.11 ± 150.06 ^bc^	1062.41 ± 51.03 ^c^	1250.78 ± 55.99 ^a^
Na	513.43 ± 190.50 ^a^	304.48 ± 337.47 ^b^	453.17 ± 103.80 ^ab^	319.04 ± 53.19 ^ab^	254.08 ± 208.22 ^ab^	227.21 ± 71.97 ^b^	405.53 ± 162.88 ^ab^
Se	0.29 ± 0.17 ^a^	0.09 ± 0.09 ^b^	0.30 ± 0.06 ^a^	0.15 ± 0.09 ^ab^	0.17 ± 0.09 ^ab^	0.11 ± 0.05 ^ab^	0.28 ± 0.04 ^a^
Sr	4.02 ± 1.90 ^a^	1.81 ± 1.17 ^ab^	4.31 ± 1.13^a^	1.46 ± 1.07 ^ab^	2.55 ± 2.88 ^ab^	0.97 ± 0.38 ^b^	4.57 ± 1.03 ^a^
Zn	58.90 ± 3.88 ^b^	64.50 ± 7.74 ^b^	61.35 ± 3.63 ^b^	80.34 ± 4.03 ^a^	65.03 ± 10.42 ^ab^	64.44 ± 7.14 ^b^	57.26 ± 2.68 ^b^

Different superscript letters within a row indicate a significant difference for each cultivation method (*p* < 0.05). The units for protein and amino acids are expressed as g/100 g, potassium content is expressed as g/kg, and the units for other mineral elements are expressed as mg/kg. Ala: alanine; Arg: arginine; Asp: aspartic acid; Glu: glutamic acid; Gly: glycine; His: histidine; Ile: isoleucine; Leu: leucine; Lys: lysine; Met: methionine; Phe: phenylalanine; Pro: proline; Ser: serine; Thr: threonine; Tyr: tyrosine; Val: valine; EAA: essential amino acid; TAA: total amino acid.

**Table 2:** Mean, standard deviation, and median of δ^13^C, δ^15^N, δ^2^H and δ^18^O in *Volvariella volvacea* from different origins.

Origins	*δ*^13^C/‰		*δ*^15^N/‰		*δ*^2^H/‰		*δ*^18^O/‰	
	Mean ± SD	Median	Mean ± SD	Median	Mean ± SD	Median	Mean ± SD	Median
Fujian	−10.98 ± 2.67 ^a^	−9.70	7.38 ± 4.47 ^a^	8.74	−15.20 ± 5.27 ^ab^	−18.29	18.28 ± 0.93 ^b^	18.46
Guangdong	−23.41 ± 0.43 ^c^	−23.20	0.36 ± 2.56 ^c^	−0.64	−16.98 ± 4.45 ^ab^	−17.76	19.92 ± 1.74 ^ab^	20.57
Hubei	−9.70 ± 0.22 ^ab^	−9.74	8.53 ± 1.59 ^ab^	7.95	−16.88 ± 5.04 ^ab^	−18.54	17.96 ± 0.93 ^ab^	18.34
Jiangsu	−15.46 ± 1.61 ^bc^	−15.34	1.32 ± 1.87 ^c^	0.96	−15.02 ± 5.64 ^ab^	−14.22	19.37 ± 1.29 ^ab^	19.47
Jiangxi	−10.57 ± 2.35 ^a^	−9.51	8.89 ± 4.29 ^a^	9.84	−17.23 ± 3.63 ^ab^	−18.26	19.05 ± 1.27 ^ab^	18.51
Shanghai	−19.48 ± 3.61 ^c^	−19.28	1.48 ± 1.98 ^bc^	1.17	−12.92 ± 5.11 ^a^	−11.60	19.84 ± 1.74 ^a^	19.74
Zhejiang	−9.72 ± 0.55 ^a^	−9.60	8.51 ± 1.54 ^a^	8.43	−17.75 ± 4.00 ^b^	−17.77	18.29 ± 0.83 ^ab^	18.23

SD: Standard deviation. Different superscript letters within a column indicate a significant difference for each cultivation method (*p* < 0.05).

**Table 3:** Geographical origin traceability models of *Volvariella volvacea*.

Geographic Origin	Accuracy/%	Total Accuracy/%	The VIP Order
Guangdong–Jiangsu–Shanghai	98.70	93.60	*δ*^15^N > *δ*^13^C > 1 > *δ*^18^O > *δ*^2^H
Fujian–Hubei–Jiangxi–Zhejiang	85.42		
Guangdong	100	100.00	*δ*^13^C > 1 > *δ*^15^N > *δ*^18^O > *δ*^2^H
Jiangsu	100		
Guangdong	63.64	86.96	*δ*^13^C > *δ*^2^H > 1 > *δ*^15^N > *δ*^18^O
Shanghai	94.29		
Jiangsu	93.55	81.82	*δ*^13^C > 1 > *δ*^2^H > *δ*^18^O > *δ*^15^N
Shanghai	71.43		
Fujian	100	80.77	*δ*^13^C > 1 > *δ*^18^O > *δ*^2^H > *δ*^15^N
Hubei	0		
Fujian	100.00	74.19	*δ*^18^O > 1 > *δ*^2^H > *δ*^15^N > *δ*^13^C
Jiangxi	20.00		
Fujian	85.71	63.64	*δ*^13^C > *δ*^2^H > 1 > *δ*^15^N > *δ*^18^O
Zhejiang	25.00		
Hubei	60.00	86.67	*δ*^18^O> 1 > *δ*^13^C > *δ*^15^N > *δ*^2^H
Jiangxi	100.00		
Hubei	40.00	76.47	*δ*^18^O > 1 > *δ*^2^H > *δ*^13^C > *δ*^15^N
Zhejiang	91.67		
Jiangxi	60.00	68.18	*δ*^18^O > 1 > *δ*^13^C > *δ*^2^H > *δ*^15^N
Zhejiang	75.00		

Accuracy/% = CC/N × 100; Total accuracy/% = (CC1 + CC2)/(N1 + N2) × 100. Correct Class (CC): The number of a specific class correctly classified; N: the total number of a specific class; CC1 and CC2: the number of samples correctly classified in each of the two classes; N1 and N2: the total number of samples in each class. VIP: variable importance in projection.

## Data Availability

The original contributions presented in the study are included in the article, further inquiries can be directed to the corresponding author.

## Abbreviations

The following abbreviations are used in this manuscript:

Ala	alanine
ANOVA	analysis of variance
Arg	arginine
Asp	aspartic acid
EAA	essential amino acids
FAO	the United Nations Food and Agriculture Organization
FHJZ	Fujian, Hubei, Jiangxi, Zhejiang
GJS	Guangdong, Jiangsu, Shanghai
Glu	glutamic acid
Gly	glycine
His	histidine
Ile	isoleucine
Leu	leucine
Lys	lysine
Met	methionine
PCA	principal component analysis
Phe	phenylalanine
PLS-DA	partial least squares discriminant analysis
Pro	proline
Ser	serine
TAA	total amino acids
Thr	threonine
Tyr	tyrosine
Val	valine
VIP	Variable importance in projection
